# Homoharringtonine inhibits fibroblasts proliferation, extracellular matrix production and reduces surgery-induced knee arthrofibrosis via PI3K/AKT/mTOR pathway-mediated apoptosis

**DOI:** 10.1186/s13018-020-02150-2

**Published:** 2021-01-06

**Authors:** Yu Sun, Jihang Dai, Rui Jiao, Qing Jiang, Jingcheng Wang

**Affiliations:** 1 Department of Orthopedics, Clinical medical college of Yangzhou University, Northern Jiangsu People’s Hospital, Yangzhou, 225001 Jiangsu China; 2 School of Medicine, Nanjing University, Nanjing, 210008 Jiangsu China; 3 Dalian medical university, Dalian, 116044 Liaoning China

**Keywords:** Homoharringtonine, Fibroblasts proliferation, Fibroblast apoptosis, Intraarticular fibrosis, PI3K/AKT/mTOR signaling pathway

## Abstract

**Background:**

The prevention of surgery-induced intraarticular fibrosis remains a challenge following orthopedic surgery. Homoharringtonine (HHT) has been reported to have positive effects in preventing various kinds of fibrosis. However, little is known regarding its effect as well as the potential mechanism of HHT in preventing surgery-induced intraarticular fibrosis.

**Methods:**

Various concentrations of HHTs were locally applied in vivo to reduce knee intraarticular fibrosis in rabbits. Histological macroscopic assessments such as hematoxylin and eosin (HE) staining, Masson’s trichrome staining, and Picric-sirius red polarized light were used to evaluate the effect of HHT in reducing intraarticular fibrosis. CCK-8, cell cycle assay, and EdU incorporation assay were used in vitro to detect HHT’s effect on inhibiting fibroblast viability and proliferation. The effect of HHT on fibroblast differentiation, extracellular matrix production, and apoptosis were evaluated by western blot, flow cytometry, immunofluorescent staining, and TUNEL analysis. Moreover, the expressions of PI3K/AKT/mTOR signaling pathway were detected.

**Results:**

The results demonstrated that HHT could reduce the formation of intraarticular fibrosis. HHT was also found to induce fibroblast apoptotic cell death in a dose- and time-dependent manner in vitro. Moreover, HHT could effectively inhibit the production of the extracellular matrix secreted by fibroblasts and inhibited the expression of p-PI3K, p-AKT, and p-mTOR in a dose-dependent manner. After treating with insulin-like growth factor-1 (IGF-1), an activator of the PI3K/AKT axis, the expressions of pro-apoptosis-related proteins were decreased, and the fibroblast apoptosis rate was also inhibited.

**Conclusions:**

In conclusion, this study demonstrated that HHT could reduce the formation of intraarticular fibrosis through the inhibition of fibroblast proliferation, extracellular matrix production, and the induction of fibroblast apoptotic cell death. Furthermore, its potential mechanism may be through the suppression of the PI3K/AKT/mTOR signaling pathway.

## Background

Excessive intraarticular fibrosis of the knee is a serious complication that can occur following knee surgery [1, 2]. Although improvements in surgical techniques have reduced the incidence of many complications, outcomes of surgical treatment remain unsatisfactory [3, 4]. For many years, numerous studies have been conducted for the prevention of the formation of intraarticular scar adhesion in the knee joint. Many strategies, including arthroscopically assisted knee arthrolysis, injection of drugs into the knee joint, and functional exercise of the knee joint were used for this regard [5, 6]. However, such methods have limitations and side effects; hence, a reasonable, effective, and simple method conferring little side effects to prevent intraarticular fibrosis scar adhesion is urgently needed.

Fibroblast proliferation was considered to be one of the most important factors in fibrosis scar adhesion formation [7]. Recently, numerous studies have shown that various anti-proliferative drugs, such as hydroxycamptothecin and mitomycin C, are satisfactory in reducing fibrosis scar adhesion through the inhibition of fibroblast proliferation [8]. Moreover, certain studies have illustrated that effective prevention of fibrosis scar adhesion could be achieved through the promotion of fibroblast apoptosis [9].

Homoharringtonine (HHT), native to south China, is a kind of ancient drug used to treat hematonosis [10]. Since 2012, HHT has been approved by the American Food and Drug Administration (FDA) for the treatment of chronic myelogenous leukemia (CML). Recently, HHT was shown to be effective in inhibiting cell proliferation in certain types of cells, and the pro-apoptotic effect of HHT on specific tumor cells was also frequently reported [11]. Some reports have also demonstrated that low dose HHT could effectively reduce fibrosis scar adhesion after glaucoma filtering surgery in humans [12].

Past studies have shown that the PI3K/AKT/mTOR signaling pathway is broadly involved in fibroblast proliferation and fibronectin expression [13]. The blockage of the mTOR signaling pathway may inhibit cell proliferation and induce apoptosis; however, no reports concerning whether HHT inhibits this signaling pathway and induces fibroblast apoptotic cell death to prevent intraarticular fibrosis exist.

Does HHT have a positive effect in reducing surgery-induced intraarticular fibrosis scar adhesion? What is the role of the PI3K/AKT/mTOR signaling pathway in HHT-induced intraarticular fibrosis reduction? The present study attempts to investigate these questions and suggests a new, effective, and simple technique in treating intraarticular fibrosis in the future.

## Materials and methods

### Reagent

Homoharringtonine (HHT) was purchased from Dalian Meilun Biotechnology Co., Ltd. The purity of HHT was 98%.

### Fibroblast collection and treatment

A cell line of fibroblasts was purchased from Jenino Biotech Co., 92 Ltd. (Guangzhou, China). Then, fibroblasts were cultured in medium (DMEM; Gibco, Grand Island, NY, USA) containing 12% fetal bovine serum (Clark-BioscienceClark-Bioscience, CA, USA), 100 U/ml penicillin G, and 100 U/ml streptomycin (PS; Thermo, Rockford, IL, USA) in a stable environment (37 °C and 5% CO2). The medium was changed every third day, and cells between passages 3–5 were used in each treatment. When the cell density reached 60–70%, fibroblasts were cultured in medium without fetal bovine serum, after which every treatment was applied to the cells.

### Cell activity detection

To detect the inhibitory effect of HHT on fibroblast proliferation, a CCK-8 assay was performed. Briefly, fibroblasts were seeded into a 96-well microtiter plate in triplicate. Cells were cultured overnight, after which various doses of HHT (0, 0.5, 1, 2, 4, 8 μg/ml) were applied to the cells. Moreover, fibroblasts were treated with 2 μg/ml HHT for different durations. According to the instructions, 10 μl CCK-8 was added to each well of the plates for 1.5 h at 37 °C. Cells stained with CCK-8 were considered to be positive. The rate of fibroblast activity was calculated according to the instructions.

### Cell cycle analysis

Essentially, fibroblasts were first inoculated into six orifice plates for overnight culture. The control group was treated with serum-free medium. Subsequently, cells were collected from all control groups and treated with 2 μg/ml HHT for 24 h. Next, all cells were immobilized with 75% ethanol, and the cells were suspended in 0.5 ml propidium iodide for 20 min. The cell distribution at different cycles was analyzed by the ModFit software, including g0/g1, s, and g2/m.

### DNA synthesis analysis of fibroblasts

We evaluated the effect of HHT on fibroblast proliferation using cell light Kfluor488 EDU kit (Ribbio, Guangzhou, China). After HHT intervention, 50 μm of 5-ethynyl-2′-deoxyuridine (EDU) were added to each Petri dish. Two hours later, it was fixed with 4% polyformaldehyde for 15 min and permeated with 0.5% Triton X-100 for 15 min. The fibroblast nuclei were then stained with 4′,6-diamidino-2-phenylindole (DAPI). Finally, the positive staining results of EDU were observed by inverted fluorescence microscopy.

### Flow cytometry analysis of fibroblast apoptosis

Annexin V-FITC/PI double labeling (Becton, Dickinson, CA, USA) was used to test for fibroblast apoptosis. Following treatment with different doses (0, 1, 2, and 4 μg/ml) HHT for 24 h) of HHT for 24 h in the wells, the cells were collected and washed with cold PBS buffer three times. The cells were resuspended in 1 ml 1 × binding buffer from which 100 μl solution was transferred to a tube. Then, according to the instructions, 5 μl PI along with FITC Annexin V were added to each tube. Following incubation by FITC Annexin V and PI for 15–20 min in the condition of protection from light at room temperature and with the additional 1 × binding buffer of 400 μl, the mixture was detected by flow cytometry.

### TUNEL assay in fibroblasts in vitro and in vivo

Apoptosis of fibroblasts was detected by the TUNEL test system (KeyGEN, Nanjing, China). Fibroblasts were cultured in vitro on a glass slide in a 6-well plate overnight. After treatment of HHT (0, 1, 2, and 4 μg/ml) for 24 h, cells were fixed with 4% paraformaldehyde solution for 20 min. After staining according to the instructions, TUNEL-positive cells were detected via fluorescence microscope. TUNEL-positive fibroblasts were stained green, and all fibroblast nuclei were stained with DAPI, which appeared blue. In regard to the TUNEL assay on fibroblasts in vivo, the sections of intraarticular fibrosis scar tissue were deparaffined at 60 °C for 1 h and rehydrated using gradient ethanol. Then, the sections were stained according to the instructions. The images were captured via fluorescence microscope.

### Immunofluorescence detection

After 24 h of 2 μg/ml HHT treatment, the fibroblasts were fixed with 4% paraformaldehyde for 10 min and treated with 0.1% Triton X-100 at room temperature for 20 min. Anti-alpha smooth muscle actin (α-SMA), anti-collagen I and collagen III, and anti-cleaved-caspase3 antibody ((1:100; Abcam, Shanghai, China) were then used to incubate the cells overnight at 4 °C. After full cleaning, anti-rabbit IgG antibody (AB150078, ABCAM) (1:100 dilution) was utilized. Following incubation at room temperature for 1.5 h, the nuclei were stained with DAPI for about 15 min. Finally, the samples were examined under a fluorescence microscope and confocal laser scanning microscope.

### Western blot assay

Fibroblasts were harvested after various treatments. Radioimmuno-precipitation assay (RIPA) buffer (Beyotime, Hangzhou, China) was used to lyse cells on ice, and BCA Protein Detection Kit (Beyotime, Hangzhou, China) was used in protein quantification. Protein lysate of different groups was separated by western blot at 8–15% SDS-PAGE. Then, PVDF membranes (Millipore, Bedford, MA, USA) were soaked in the blocking buffer for 2 h at room temperature, and the membranes were incubated with primary and secondary antibodies. Last, protein bands were detected by a professional imaging system (Hewlett-Packard, Palo Alto, USA). All antibodies used in the western blot assay were purchased from Cell Signaling Technology (CST, Beverly, MA, USA).

### Animals

In this study, 36 New Zealand, white, male rabbits weighing 3500–4000 g were purchased from the experimental animal center of Yangzhou University (Yangzhou, China). The present animal study was approved by the Animal Ethics Committee of Yangzhou University, and all rabbits were treated with meticulous care. The rabbits were randomly split into three groups (12 rabbits per group).

### Rabbit model and local application of HHT

Rabbit knee intraarticular adhesion models were built in line with previous studies [14, 15]. Accordingly, after giving anesthesia with 0.7% pentobarbital sodium (6 ml/kg body weight) via intravenous injection, the fur was shaved around the left knee joint, after which the joint capsule was exposed via knee medial incision. Then, a complete 0.5 × 0.5 cm area of the bone cortex was removed from both sides of the femoral condyle. The cartilage particulars were observed to be intact. Following satisfactory hemostasis around the operative region, cotton pads (0.4 × 0.4 cm) with different concentrations of HHT (0.2 mg/ml, 0.1 mg/ml, and saline) were applied to the femoral condyle defect areas for 10 min. Furthermore, a wet gauze was used to curtail the side effects of HHT on the surrounding tissues. Ten minutes later, the surgery sites were immediately douched with saline to remove the remaining drugs.

### Histological analysis

The other six rabbits were picked 4 weeks following the surgeries. The total surgical knee joint was removed with tissues around and fixed in 10% buffered formalin. One week later, all samples were soaked in ethylenediamine tetraacetic acid (EDTA) for 3 weeks for decalcification. All sections were obtained from the samples embedded by paraffin. The sections of each group were stained with hematoxylin and eosin(HE)and Masson’s trichrome stains. Images of the intraarticular fibrosis scar adhesion were captured using a microscope (UVIGAS7001B, UK) at a magnification of × 40. Three counting fields (approximately 100 × 100 mm each) of the intraarticular adhesion sites were randomly selected to count the number of fibroblasts at a magnification of × 200.

### Analysis of hydroxyproline content (HPC)

Previous studies have suggested that HPC assay was satisfactory in detecting the amount of collagen in scar tissue [14]. Approximately 20 mg wet-weight scar tissue was taken from femoral condyle defect sites following macroscopic evaluation. After lyophilizing, grounding, hydrolyzing, and neutralizing step-by-step, the scar tissues were incubated with chloramine T for 30 min. Then, 1 ml hydroxyproline developer was added to the samples and standards. The absorbance was detected using a spectrophotometer at 558 nm. The HPC/mg scar tissue was computed based on the typical curve built with the serial concentrations of commercial hydroxyproline.

### Sirius picrate red polarized light method

First, all sections were dehydrated with gradient alcohol and treated with Sirius bright red dye for an hour. They were then rinsed thoroughly using tap water three times, treated with hematoxylin dye for 8–10 min, and rinsed with tap water again for 10 min. Finally, the slices were dehydrated with gradient ethanol and sealed with xylene. The sections were observed under a polarizing microscope to distinguish type I collagen from type III collagen.

### Statistical analysis

The statistical analysis of all the data from this study was done on the SPSS 19.0 statistical software. The data were expressed as mean ± standard deviation, and statistical significance was defined as a *P* value < 0.05.

## Results

### HHT reduced intraarticular fibrosis after knee surgery in rabbits

**Fig. 1:**
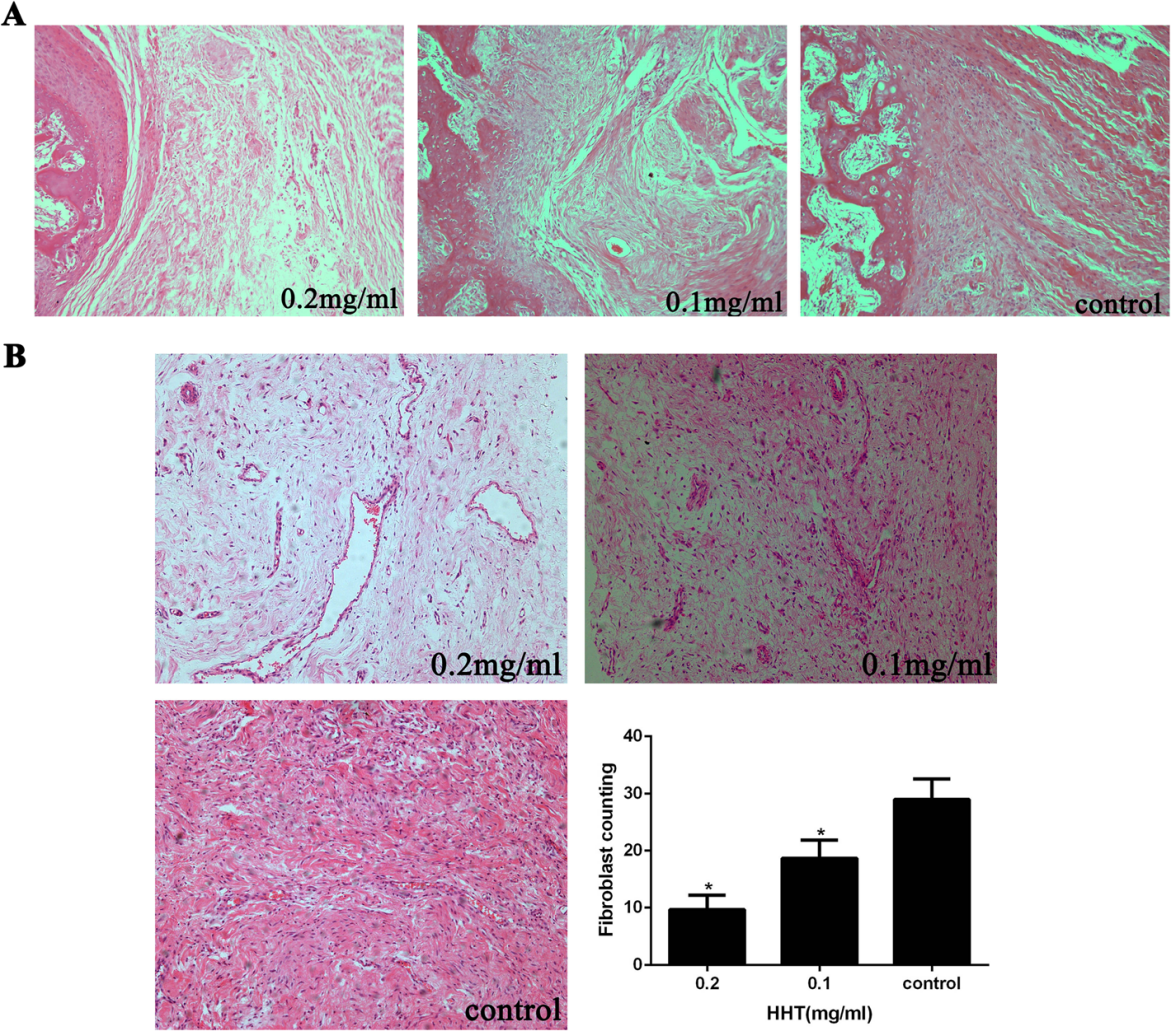
Histological analysis images of intraarticular fibrosis tissue. **a** Loose or thin fibrosis scar tissues with few adhesions to bone cortex were found in 0.2 mg/ml HHT-treated group. Moderate fibrosis scar tissues with slight adherence to bone cortex were found in 0.1 mg/ml HHT-treated group. Dense or thick fibrosis scar tissues with extensive or firm adherence to bone cortex were found in control group. All sections were stained by the mean of H&E, and the magnification was × 40. **b** In vivo, the images of fibroblasts in intraarticular fibrosis scar tissues of each group. The magnification was × 200. Three counting areas were selected from every section to calculate fibroblast number. *P < 0.05 versus the control group

To explore the effects of HHT in reducing intraarticular fibrosis, different concentrations of HHT were applied to the femoral condyle defects. The H&E images demonstrated that extensive and dense fibrosis was found around the decorticated sites of the femoral condyle in the control group. Following application of HHT, moderate intraarticular fibrosis was found in the 0.1 mg/ml HHT-treated group, while the decorticated areas were covered with loose or thin fibrotic tissues in the 0.2 mg/ml HHT-treated group (Fig. 1a).

Following the treatment of HHT, the fibroblast density gradually decreased compared to that of the control group. The fibroblast count in the fibrotic tissue around the decorticated sites in the 0.2 mg/ml HHT-treated group was significantly lower than that in the 0.1 mg/ml HHT-treated and control groups (Fig. 1b).

**Fig. 2:**
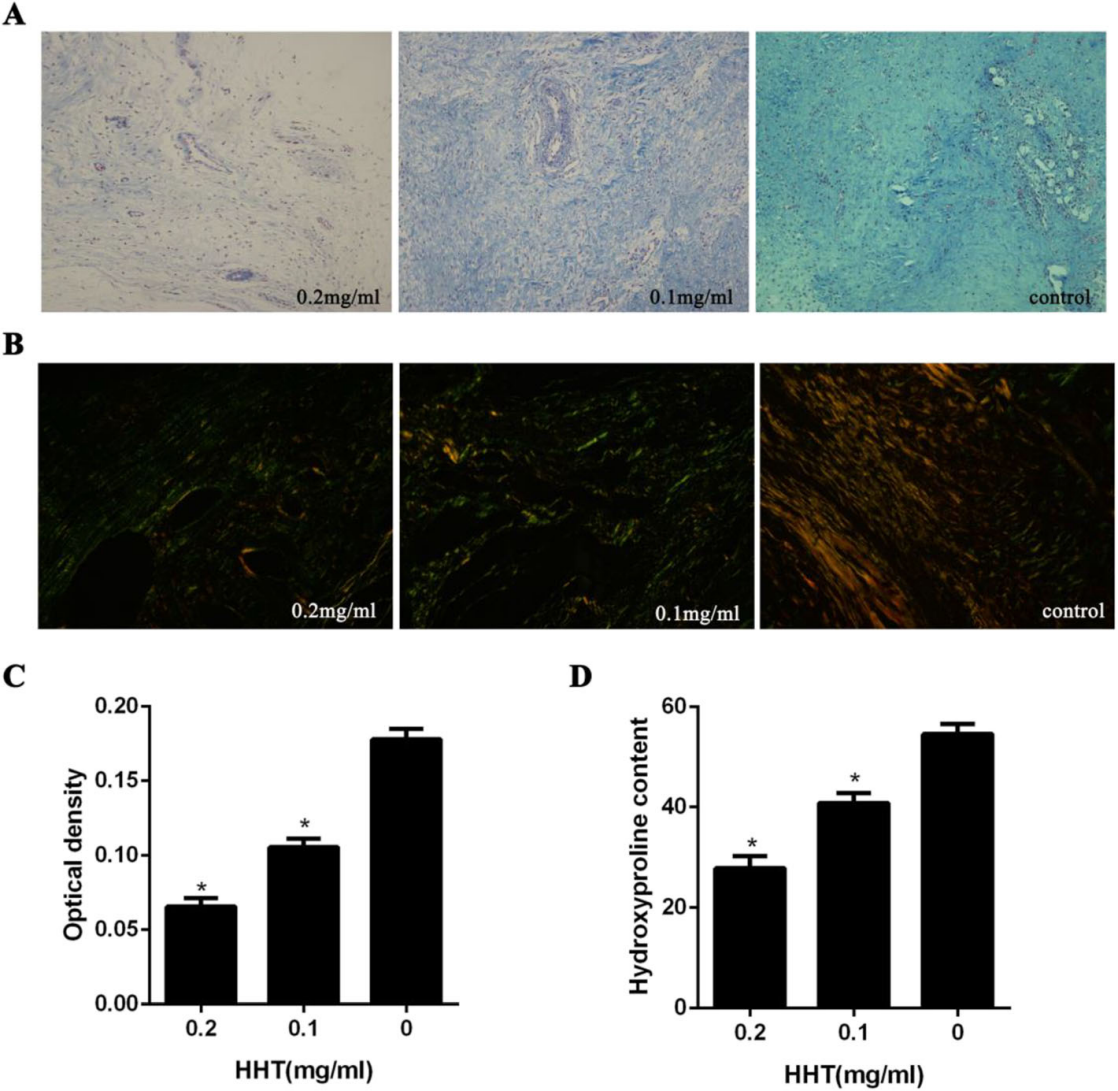
Collagen analysis of intraarticular fibrosis scar tissues treated with 0.2 mg/ml, 0.1 mg/ml HHT and saline. **a** As the sections were stained with Masson’s trichrome, intraarticular collagen tissues appeared blue. And the magnification was × 200. HHT suppressed epidural collagen synthesis and fibrosis in a dose-dependent manner. **b** Under polarized light microscopy (× 400), type I collagen was red or yellow, and type III collagen was green. With the increase of drug concentration, the content of type I collagen fibers in intra-articular fibrosis decreased significantly. **c** Intraarticular collagen optical density value of each group. *P < 0.05 compared with the value in control group. **d** HPC was expressed as μg/mg. The amount of hydroxyproline was decreased with the rising concentration of HHT

To detect the effect of HHT on intraarticular collagen synthesis, Masson’s trichrome staining was performed. As shown in Fig. 2a, the high density of intraarticular collagen tissue at decorticated sites was observed in the control group, while intraarticular collagen synthesis gradually decreased following treatment with HHT.

The optical density values of collagen tissue in the intraarticular fibrosis tissue of each treatment group are shown in Fig. 2c. The optical density value of collagen tissue in the 0.2 mg/ml HHT-treated group was significantly less than that in the 0.1 mg/ml HHT-treated and control groups.

In regard to Sirius scarlet staining, type I collagen was observed to be bright red or strong orange yellow, while type III collagen was green. The density of type I collagen in the control group was dense, while the content of collagen I was found to significantly decrease following treatment with HHT (Fig. 2b). The results of collagen density in the HHT-treated groups coincided with hematoxylin and eosin staining. Furthermore, HPC analysis was performed to reflect the amount of collagen in intraarticular fibrosis scar tissue. As shown in Fig. 2d, following treatment of HHT, the HPCs of intraarticular scar tissue were significantly decreased compared to that of the control group.

The observed results indicated that HHT could inhibit fibroblast proliferation and collagen production, reducing intraarticular fibrosis after knee surgery in rabbits.

### The inhibitory effect of HHT on fibroblast proliferation

**Fig. 3:**
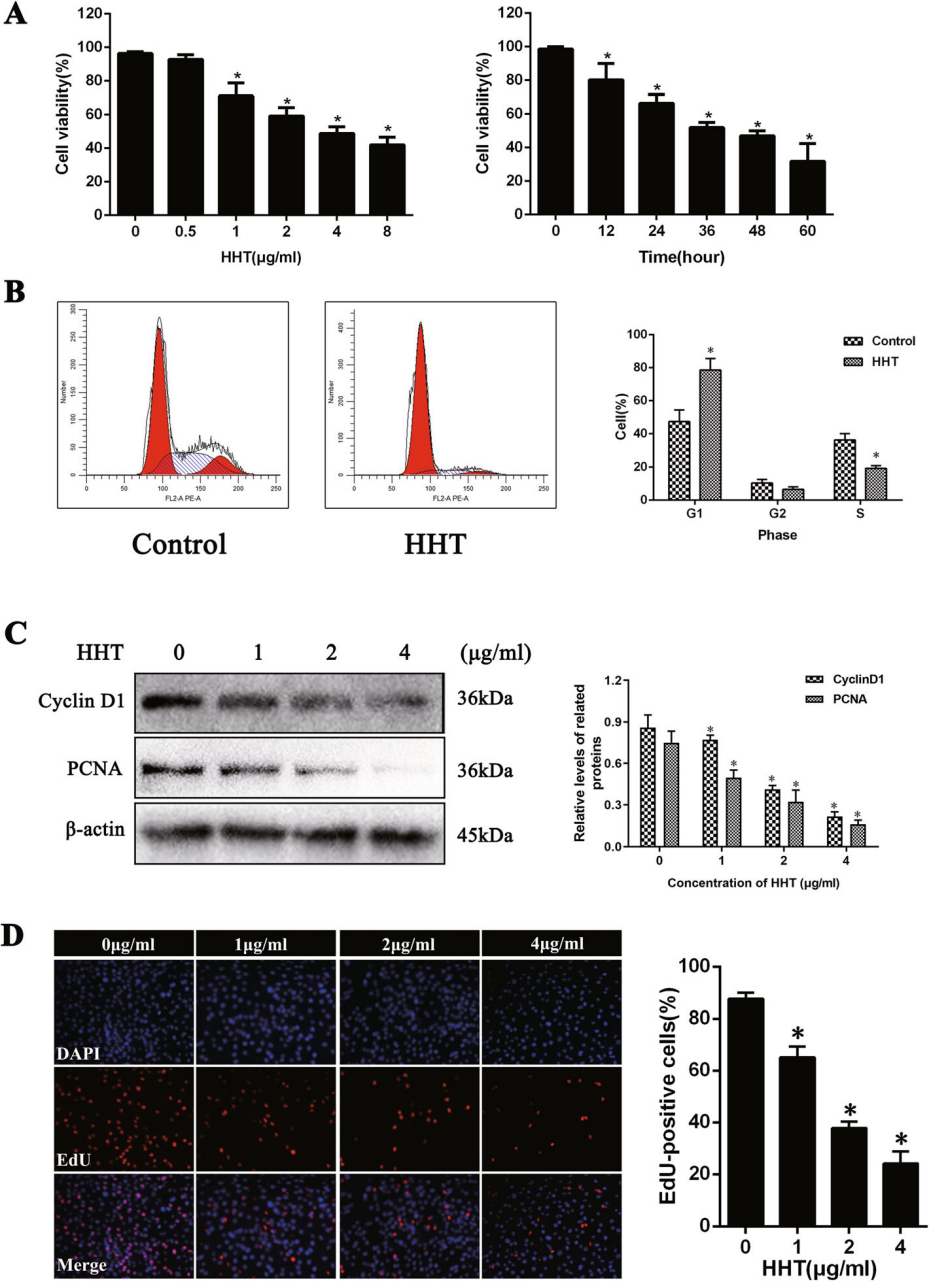
HHT inhibited fibroblast proliferation in vitro. **a** In vitro, cell viability was detected via CCK-8 assay. HHT induced dose- and time-dependent inhibitory effects of fibroblast proliferation. **b** Flow cytometry showed the cells were arrested at G1 phase and the cells in the S phase were significantly decreased. **c** In vitro, western blots shown that application of HHT could reduce the expression of Cyclin D1 and PCNA, which were known as cell proliferation protein markers. β-actin was used as a control. *P < 0.05 versus the control group. **d** EDU test showed that the percentage of EDU-positive cells decreased significantly with the increase of drug concentration

To explore whether HHT inhibits fibroblast proliferation, a CCK-8 assay was used to detect the effect of HHT in regulating fibroblast viability. After being treated with HHT, fibroblast viability was observed to gradually decrease in a concentration- and time-dependent manner, indicating that HHT had a definite inhibitory effect on fibroblast viability (Fig. 3a).

The cell cycle results demonstrated that the number of fibroblasts in the G1 phase significantly increased following 2 μg/ml HHT treatment, while the number of cells in the S phase significantly decreased (Fig. 3b).

Moreover, the results of the EDU assay showed that the proliferative activity of fibroblasts significantly decreased after being treated with increasing concentrations of HHT (Fig. 3d).

To further validate the inhibitory effect of HHT on fibroblast proliferation, a western blot assay was performed to detect the expression of cell proliferation proteins such as Cyclin D1 and PCNA [16, 17]. The results demonstrated that their corresponding expression levels were significantly decreased following treatment with different concentrations of HHT (0, 1, 2, and 4 μg/ml) for 24 h in a concentration-dependent manner (Fig. 3c).

These results indicated that HHT had an inhibitory effect on fibroblast proliferation.

### Effect of HHT on fibroblast differentiation and extracellular matrix formation

**Fig. 4:**
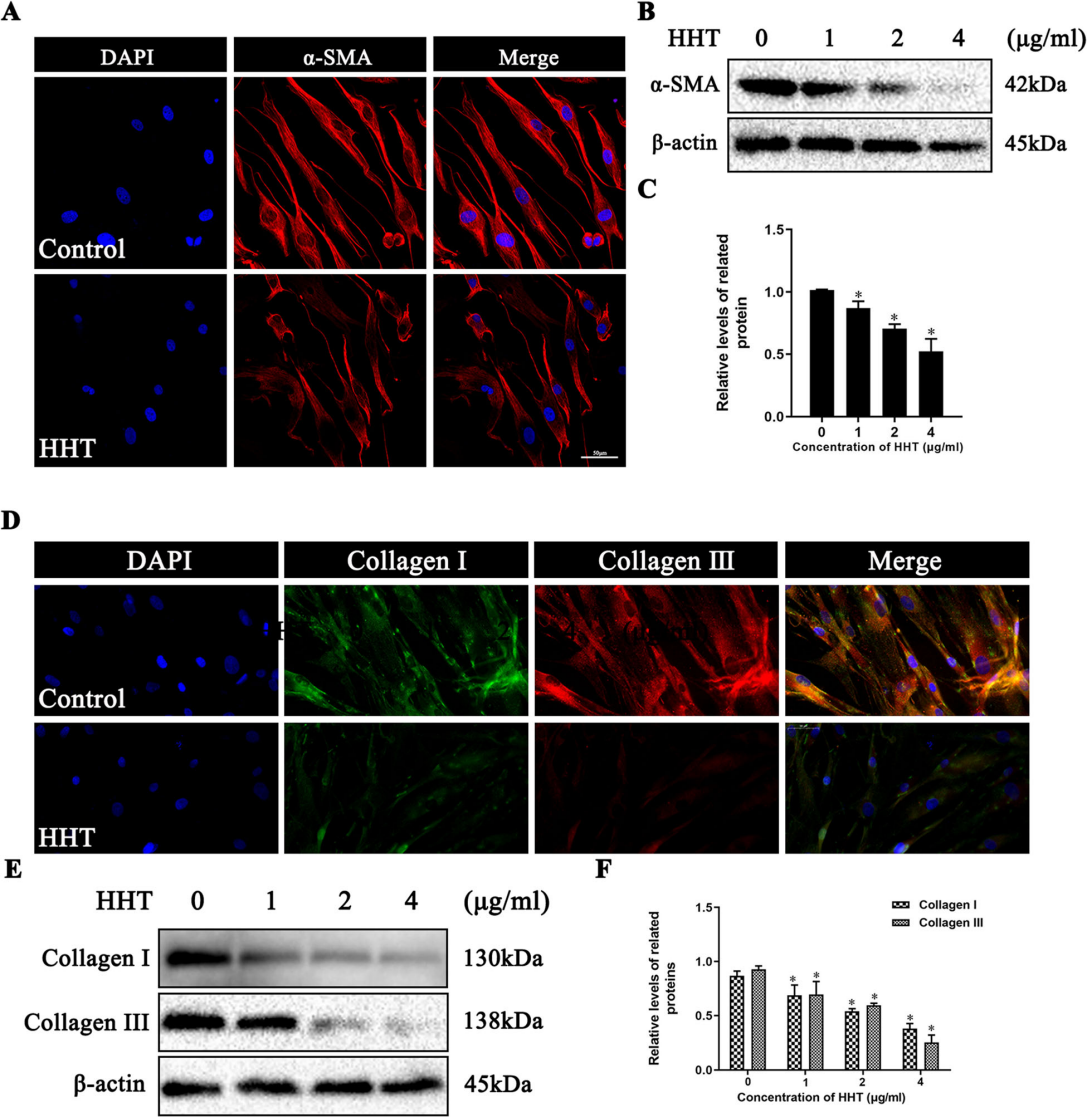
The effect of HHT on the differentiation of fibroblasts and the expression of extracellular matrix secretion. **a** After different treatment, the expression of α-SMA was detected by immunofluorescence. **b**, **c** After HHT intervention, the expression of α-SMA in fibroblasts detected by western blot assay. **d** After HHT intervention, immunofluorescence was used to detect the expression of collagen I and III. **e** Western blot analysis of collagen I and III. *Compared with the control group (*P* < 0.05)

The differentiation of fibroblasts into myofibroblasts is considered to be an important component of fibrosis. Moreover, the expression of α-SMA has been shown to be a specific marker of myofibroblasts. Therefore, the expression of α-SMA in fibroblasts was analyzed by immunofluorescence and western blot, respectively. The results showed that HHT significantly reduced α-SMA expression (Fig. 4a–c), and over formation of the extracellular matrix was observed to be an important cause of fibrosis. Type I and type III collagen are considered to be the two main types of collagen in the extracellular matrix. The expression of type I and type III collagen in fibroblasts was detected using immunofluorescence and western blot analysis. The results demonstrated that HHT could inhibit the formation of type I and III collagen in fibroblasts (Fig. 4d–f).

### HHT induced the apoptotic cell death of fibroblasts

**Fig. 5:**
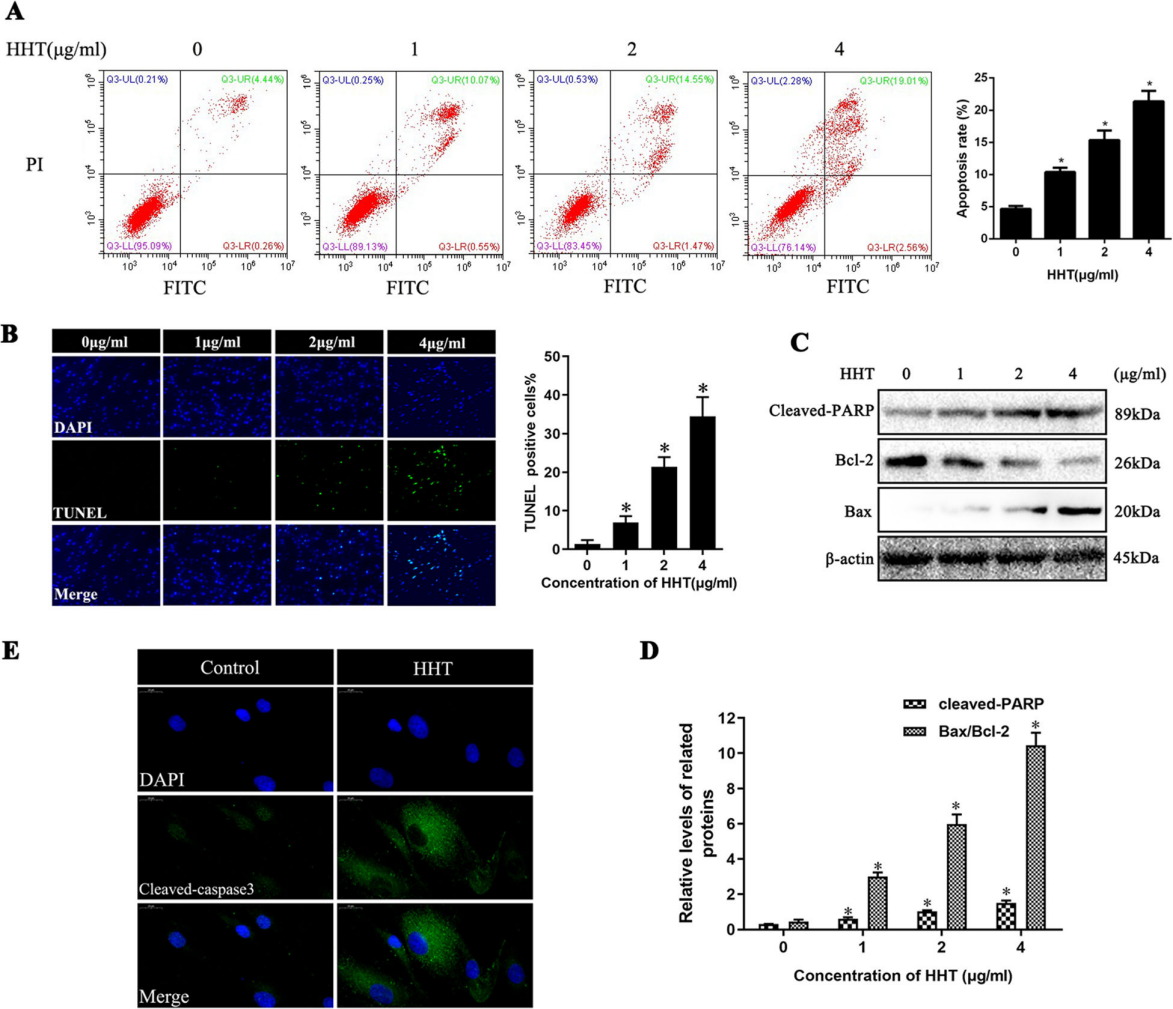
HHT induced fibroblast apoptosis in vitro. **a** In vitro, fibroblast apoptosis was detected by annexin V-FITC/PI double labeling. The result was repeated for three times. *P < 0.05 versus the control group. **b** In vitro, TUNEL staining was used to detect the pro-apoptotic effect of HHT. All nuclei of fibroblasts were blue, and the nuclei of TUNEL-positive fibroblasts were green. Three fields were picked to count the percentage of apoptotic fibroblasts in every group. *P < 0.05 versus the control group. **c**, **d** Western blots shown that application of HHT could induce the expression of cleaved-caspase3, cleaved-PARP, and Bax, which were known as pro-apoptotic proteins, while decreased the expression of Bcl-2, which was considered as an anti-apoptotic protein. β-actin was used as a control. **e** Immunofluorescence study of the anti-cleaved-caspase3 showed that the expression of cleaved-caspase3 in the cytoplasm of fibroblasts treated with 2 ug/ml HHT increased significantly 24 h later

Annexin V-FITC/PI double labeling was performed to explore whether HHT could cause fibroblast apoptosis, which was found to induce fibroblast apoptosis in a dose-dependent manner. After the application of 0, 1, 2, and 4 μg/ml HHT for 24 h, the total percentages of fibroblast apoptosis were 4.68% ± 0.47%, 10.40% ± 0.68%, 15.37% ± 1.48%, and 21.41% ± 1.65% (Fig. 5a). Furthermore, morphological examination (TUNEL assay) was performed to detect the apoptotic effect of HHT in fibroblast. After treatment with varying doses of HHT for 24 h, with the increase in concentration of HHT, TUNEL-positive cells significantly increased (*P* < 0.05) (Fig. 5b). Additionally, the western blot assay of the expression levels of pro-apoptotic protein markers (cleaved-PARP and Bax) were found to be increased with a rise in HHT dosage. However, the expression level of anti-apoptotic protein marker Bcl-2 was observed to be decreased (Fig. 5c, d). In addition, the immunofluorescence assay showed that the expression of cleaved-caspase 3, an important apoptotic marker, increased following HHT intervention (Fig. 5e).

### HHT induced fibroblast apoptosis by inhibiting the activation of the PI3K/AKT/mTOR signaling pathway

**Fig. 6:**
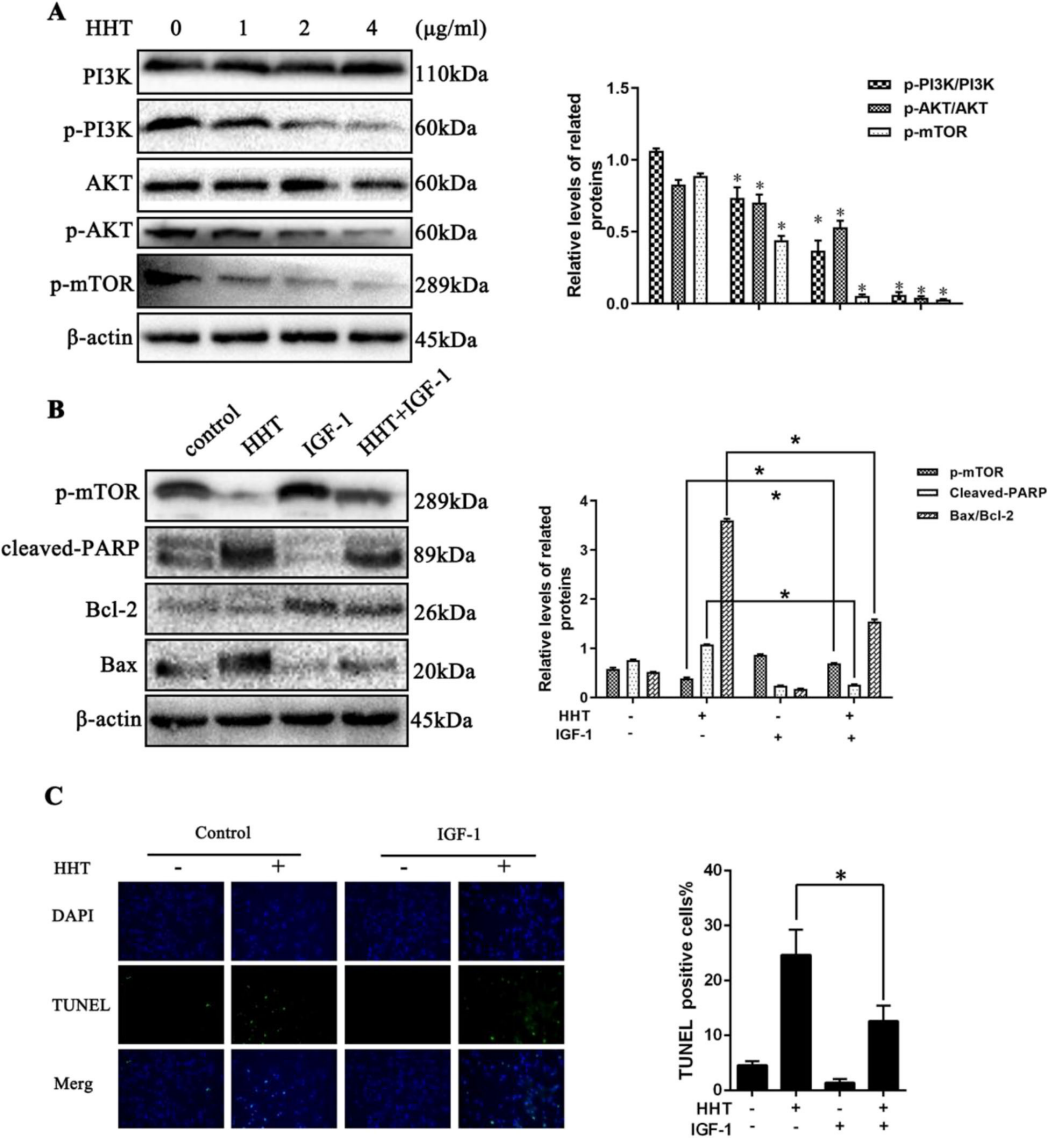
HHT induced fibroblasts apoptosis via inhibiting the activation of PI3K/AKT/mTOR signaling pathway. **a** We treated fibroblasts with different doses of HHT for 24 h. The results of western blot assay showed that the expression of phosphorylation levels of PI3K, AKT, and mTOR were significantly reduced after the application of HHT in a dose-dependent manner. **b** After the treatment of IGF-1, an activator of PI3K/AKT axis, the expressions of pro-apoptosis-related proteins were decreased, **c** and the fibroblast apoptosis rate was also suppressed

The PI3K/AKT/mTOR signaling pathway was previously verified to be involved in HHT-induced fibroblast apoptotic cell death and was reported to play a role in regulating fibroblast proliferation and apoptosis. Western blot analysis indicated that after fibroblasts were treated with the various doses of HHT (0, 1, 2, 4 μg/ml) for 24 h, the phosphorylation levels of PI3K, AKT, and mTOR were significantly reduced (Fig. 6a). IGF-1 is known to be an activator of PI3K/AKT signaling; hence, fibroblasts were separately treated with IGF-1 and HHT(1 μg/ml) as well as mixtures of HHT and IGF-1. Then, a western blot assay was used to detect the expression of the phosphorylation levels of mTOR as well as the expression levels of pro-apoptotic protein markers. Here, the expression of p-mTOR was found to be further increased, while the expression levels of pro-apoptotic protein markers were partly decreased following the treatment of IGF-1 (Fig. 6b). Furthermore, the TUNEL assay showed that the apoptosis rate of fibroblasts decreased following treatment with IGF-1 (Fig. 6c). The corresponding data suggest that HHT induces fibroblast apoptotic cell death by inhibiting the PI3K/AKT/mTOR signaling pathway.

## Discussion

Intraarticular fibrosis scar adhesion following knee surgery often causes serious complications that may lead to negative patient satisfaction [1]. Its definite pathophysiology in terms of the formation of intraarticular fibrosis scar adhesion after knee surgery has yet to be elucidated. Effective intraoperative hemostasis, postoperative chronic inflammation, and joint instability are considered to be important factors in regard to the formation and development of intraarticular fibrosis scar adhesion [18]. However, such factors would induce fibroblast proliferation, and a large number of fibroblasts may gather to surgical sites following the release of collagen fibers and evolution of fibroblasts, leading to the formation of intraarticular fibrosis scar adhesion. Thus, targeted research on fibroblasts may be beneficial in the treatment of intraarticular adhesion.

HHT is an effective component extracted from cephalotaxus and is known to be an inhibitor for protein translation [19] . Due to its special characteristics, HHT was recently found to be effective in inhibiting fibroblast proliferation, and researchers have shown that HHT possessed positive effects on proliferative eye disease [20, 21].

As an important cellular physiological process, apoptosis occurs in multicellular organisms. Recently, it was reported that promoting fibroblast apoptosis may be beneficial in reducing postoperative fibrosis scar adhesion formation. Sun et al. showed that postoperative epidural adhesion could be reduced by promoting fibroblast apoptosis [22]. Moreover, Li et al. reported that increasing fibroblast apoptosis may serve as an important mechanism underlying the reduction of intraarticular adhesion [23]. Therefore, HHT was investigated in whether it could increase the apoptosis rate of fibroblasts. Furthermore, fibroblast apoptosis’s role in the reduction of intraarticular adhesion was also examined.

In this study, both in vitro and in vivo experiments were conducted to explore whether HHT could reduce intraarticular fibrosis scar adhesion by inhibiting fibroblast proliferation and promoting fibroblast apoptosis. According to the results of postoperative macroscopic evaluation and pathological observation, this study firmly concludes that HHT could ameliorate the degree of postoperative intraarticular adhesion following local application of HHT. In regard to the in vitro studies, HHT was found to have a significant inhibitory effect on fibroblast proliferation. Furthermore, flow cytometry detection and TUNEL assay demonstrated that the application of HHT could increase the fibroblast apoptosis rate. The western blot assay done on cell pro-apoptotic protein markers (cleaved-PARP and Bax) reconfirmed that HHT could cause fibroblast apoptosis. Additionally, histopathological observation in vivo showed that HHT could markedly decrease the number of fibroblasts in intraarticular fibrosis scar tissue with a rise in HHT dosage.

After HHT was found to induce fibroblast apoptotic cell death and reduce intraarticular fibrosis, its possible mechanism was preliminarily explored. The PI3K/AKT/mTOR signaling pathway is known to play a key role in regulating fibroblast proliferation and mammalian cell apoptosis [24]. Previous studies have reported that regulating the PI3K/AKT/mTOR signaling pathway may interfere in anti-fibrotic activity. In the present study, the phosphorylation levels of PI3K, AKT, and mTOR were observed to be significantly reduced after fibroblasts were treated with HHT for 24 h. However, after treatment with PI3K/AKT activator (IGF-1), the high fibroblast apoptosis rate induced by HHT was found to be partly decreased. This signified that inhibiting of the activation of the PI3K/AKT/mTOR signaling pathway may be a mechanism of HHT in inducing fibroblast apoptotic cell death and preventing surgery-induced intraarticular fibrosis.

The effect of HHT may be reasonable explained on account of its reduction of intraarticular fibrosis scar adhesions. Essentially, following the local application of HHT in intraarticular surgery sites, the PI3K/AKT/mTOR signaling pathway was suppressed, and apoptotic cell death occurred in fibroblasts, causing the number of fibroblasts to decrease and intraarticular adhesions to eventually reduce.

The definite mechanism of adhesion formation is very complex. In the present study, the effect of HHT on fibroblast proliferation in reducing intraarticular fibrosis scar adhesion was discussed. More factors should be analyzed for future research regarding this topic. However, as an anti-tumor drug, the toxicity associated in locally applying HHT in intraarticular surgery sites remains unclear. Moreover, the side effects of HHT on other types of cells, especially chondrocytes, were not detected in this study. Therefore, this study presents a preliminary research of the effect of HHT in reducing intraarticular fibrosis scar adhesion. To this effect, additional studies should be performed in the future.

## Conclusion

The results above demonstrated that HHT could reduce intraarticular fibrosis scar adhesion by stimulating fibroblast apoptotic cell death through the suppression of the PI3K/AKT/mTOR signaling pathway.

## Data Availability

The datasets supporting the conclusions of this article are included within the article and its supplementary materials.

## Abbreviations

HHT: Homoharringtonine
HE: Hematoxylin and eosin
IGF-1: Insulin-like growth factor-1
